# A novel optimization method for hazardous materials vehicle routing with temperature-based time windows

**DOI:** 10.7717/peerj-cs.2586

**Published:** 2024-12-13

**Authors:** Lu Ding, Fangwei Zhang, Jun Ye, Fanyi Kong, Minhui Jiao

**Affiliations:** 1 School of Navigation and Shipping, Shandong Jiaotong University, Weihai, Shandong, China; 2 School of International Business, Shandong Jiaotong University, Weihai, Shandong, China; 3 School of Civil and Environmental Engineering, Ningbo University, Ningbo, Zhejiang, China

**Keywords:** Vehicle routing problem, Multi-objective optimization, Temperature-based time windows, Adaptive large neighborhood search algorithm

## Abstract

As a concrete achievement of sharing economy, sharing intermediate bulk containers (IBCs) have emerged and developed in recent years. Meanwhile, high temperature is one of the essential factors in routing optimal problems for vehicles with hazardous material (hazmat). Therefore, to address the above issue, a variant of the hazmat vehicle routing problem of sharing IBCs is proposed. Correspondingly, a mixed non-linear integer programming model is refined considering temperature-based time windows. Specifically, the given problem is solved by using a novel adaptive large neighborhood search (ALNS) algorithm. The main innovation points are as follows. Firstly, temperature-based time windows are quantified and integrated into the proposed hazmat vehicle routing optimal model. Secondly, novel heuristic operators are introduced in the ALNS algorithm. Finally, 18 numerical examples for the Solomon set demonstrate that the proposed algorithm is suitable to solve this kind of hazmat vehicle routing optimal problem.

## Introduction

Currently, as the importance of sustainable development continues to rise, there is a growing focus on maximizing resource utilization. As a novel means of transporting liquefied hazardous materials (hazmat), intermediate bulk containers (IBCs) have become increasingly prominent in the complex environment of global supply chains (Coelho et al., 2020). IBC serves as one of the important media for transporting hazmat due to its low transportation costs, so research gradually focused on hazmat transportation using it. By combining factors affecting resource utilization and shared containers, using shared IBCs is proposed as a new approach to ensure transportation sustainability and safety. Therefore, it is important for hazmat transportation to apply the sharing IBCs on the hazmat transportation.

Meanwhile, a notable increase in hazmat transportation has occurred, driven by rapid global industrialization. In the event of spills or traffic accidents, hazmat typically has a high probability of causing severe casualties and property damage (Ma, Zhou & Yang, 2020). Therefore, it is necessary to take targeted measures based on the critical risk factors in accidents to prevent similar accidents from happening again. From the analysis of causes of hazmat accidents, it is clear that the frequency of accidents is relatively high in summer cloudy weather conditions (Jaaron, Othman & Saleh, 2019). High-temperature weather is one of the potential factors affecting accidents (Asefi, Shahparvari & Chettri, 2019; Guo, Luo & Ma, 2023), and adjusting to temperature variations during transportation is a challenging problem.

Temperature variations are a crucial risk factor for various types of hazmat, which significantly impact hazardous material transportation. For example, flammable substances, like gasoline and alcohol, are prone to volatile and combustion when temperature condition increases. Toxic chemicals release hazardous fumes posing severe risks of safety, such as chlorine and ammonia. In actual situations, temperature changes are dynamic and influence the safety and route choice of hazmat transportation. Existing models typically assume constant risk factors without accounting for temperature-sensitive conditions (Lu, Yang & Yang, 2023; Niu & Ukkusuri, 2020). Therefore, this study addresses these gaps by introducing a novel approach that incorporates temperature-based time windows and a quantifiable temperature risk function. By setting temperature levels and thresholds, the model provides a method for hazmat vehicle routing problem (HVRP) under high-temperature weather. To better understand the current research progress on shared containers in the field of HVRP and its variants, as well as to optimize model construction and the application of heuristic algorithms, a review of the relevant literature was conducted.

### Literature review

Recently, scholars have conducted exploratory research on shared containers, which provides reference for this study. For example, Islam & Olsen (2014) studied truck-sharing problems in supply chain and transportation management collaboration delivery as an example of using empty containers. Then, Sterzik, Kopfer & Yun (2015) studied the transportation situation in the hinterland of seaports and verified the advantages of shared empty containers among cooperative trucking companies. Later, Tang et al. (2021) integrated the shared container technique to maximize the transfer and leasing of empty containers. They was proposed a multi-period optimization approach to increase container utilization while minimizing overall costs and ensuring a reliable transportation network. At the same time, Lin & Juan (2021) studied the empty container relocation problem based on container sharing among shipping lines, using a solution framework that considers the exchange of empty containers. Currently, the concept of sharing containers has been evolving in hazmat transportation in order to provide development opportunities, which combines the advantages of shared economy with its transportation scenario.

In the study of vehicle routing problem (VRP) and vehicle routing problem with time windows (VRPTW), multiple objective optimization (MOO) has more practical application scenarios compared to single objective optimization. Bula et al. (2019) proposed an approach for determining trade-off solutions and applied two algorithms to address the issue of conflicting objectives in heterogeneous fleets. Hu et al. (2019) proposed a novel optimization method considering multi-objectives and constraints, such as traffic restrictions and imprecise time windows. Jiang et al. (2020) suggested a novel risk assessment method considering several influencing factors, and proposed an MOO model with vehicle size and route to focus on the special constraints of heterogeneous fleets. Ouertani, Ben-Romdhane & Krichen (2022) developed a dynamic hazmat VRP choice model to reduce transportation costs and hazards within a certain time limit. Lu, Yang & Yang (2023) developed an MOO model to demonstrate the significance of weather conditions in synchronous delivery decision-making for truck-drone collaborative delivery. Wang, Wang & Zhou (2024) proposed a layered scheme incorporating crowding distance in the layered environmental process to improve the search speed of Pareto solutions. It is evident that multi-objective optimization methods, which incorporate a variety of factors, hold significant potential for development—such as considerations of traffic flow, heterogeneous fleets, and uncertain time windows. However, existing studies primarily focus on optimizing personnel and management within the transportation process, which limits research that incorporates environmental factors. Thus, it is necessary to investigate the key factors affecting the cost, efficiency and safety of hazmat transportation.

In addition, the adaptive large neighborhood search (ALNS) algorithm, which was proposed by Ropke & Pisinger (2006), is selected to solve problem. Usually, the ALNS algorithm is rooted in metaheuristic algorithms and swarm intelligence algorithms (Baños et al., 2013; Shao et al., 2017; Rego et al., 2022). Related to this research, Chen et al. (2018) proposed a novel ALNS algorithm that satisfies VRPTW constraints, and improved solution quality by checking the feasibility of inserting customer nodes. Alinaghian & Shokouhi (2018) introduced a hybrid ALNS algorithm approach which solves the multi-depot multi-room VRP rapidly, where different vehicles meet the same customer demand. Shen et al. (2020) proposed a hybrid algorithm by using multiple algorithms to solve VRPTW. This approach improved the convergence speed of the model and resulted in high-quality solutions. Wen, Sun & Yu (2022) solved the multi-depot green VRP by using an improved ALNS algorithm. The algorithm increased neighborhood diversity by introducing novel heuristic operators, which reduced iteration time. Erdogan & MHallah (2023) proposed an ALNS algorithm using variable neighborhood search with simultaneous delivery and shared installation. It is visible that the ALNS algorithm is widely applied to find the global optimal solution to optimization problems. Its greatest advantage lies in its ability to navigate more easily from one neighborhood to another, discovering better regions within a strictly constrained solution space, thereby demonstrating strong global search capabilities (Mara et al., 2022).

### Motivations and contribution

Considering the influence of weather on the hazmat transportation process, a hazmat vehicle routing problem with temperature-based time windows (HVRP-TTW) in port chemical park is introduced. The motivation is to give a method to ensure the safe, economical and efficient transportation of hazmat in high-temperature weather. Specifically, the main contributions of the study are as follows.

(1) This study introduces a sharing method that uses IBCs as a medium for hazmat transportation, which is shared across any customer node. This approach reduces resource usage, and fosters enterprise cooperation in supply chain management.

(2) This study presents a temperature-based time window in the model of hazmat vehicle routing planning to adjust the service time associated with high-temperature weather. Meanwhile, by analyzing the influence of a high-temperature weather on the incident probability, the risk associated with temperature fluctuations is quantified in the model.

(3) This study proposes a novel set of heuristic operators that quantify the importance of customer nodes by considering factors such as service time windows, transportation distances, and customer demand. The goal is to expand the search range of neighborhood solutions by utilizing various destruction and repair operators.

**Figure 1:**
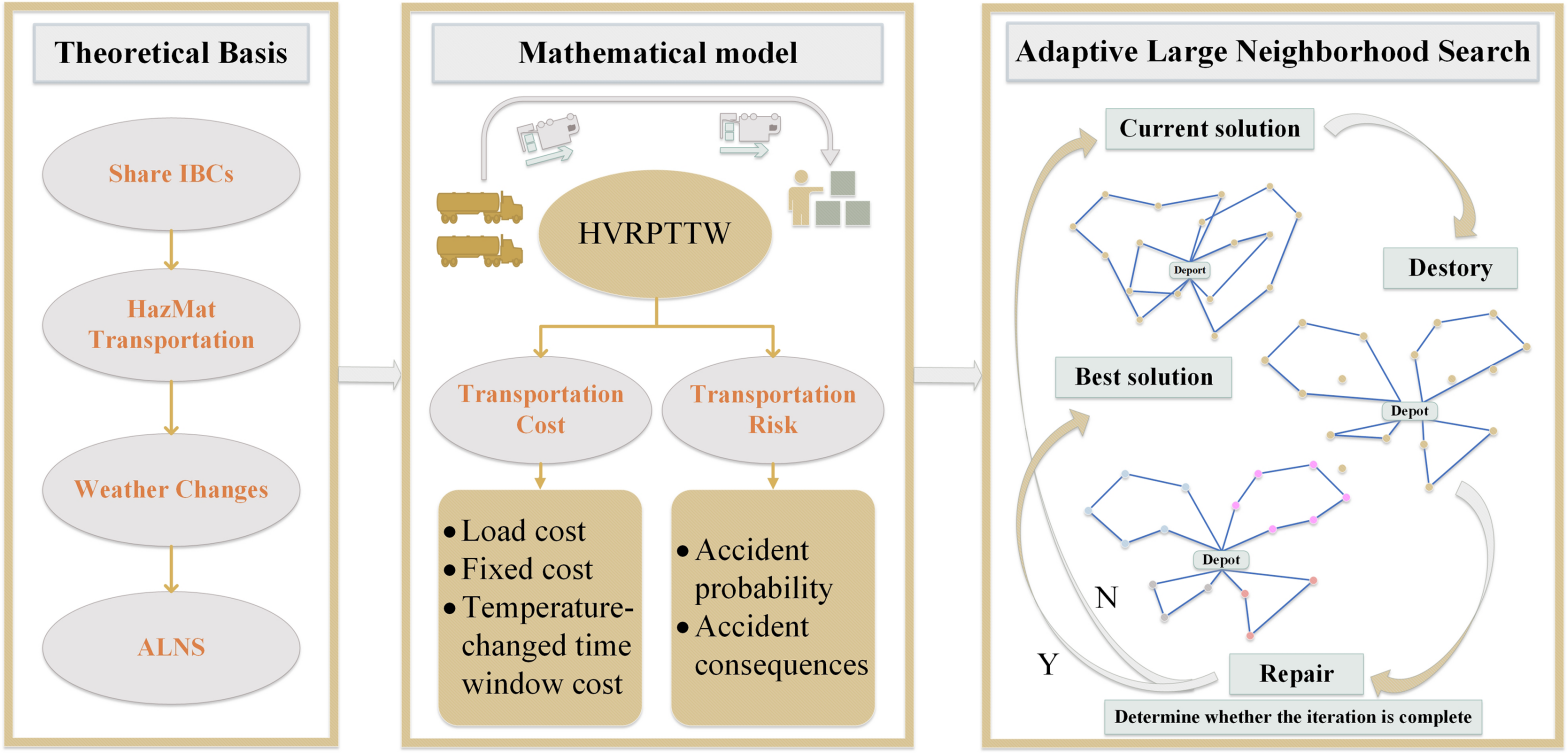
The primary processes of this study.

The remainder is organized as follows. “Materials and Methods” introduces problem definition, notations and the proposed model. “Algorithm and Model Solution Procedure” proposes the process of the improved ALNS algorithm. To verify the effectiveness of the ALNS algorithm, “Results and Discussion” introduces a comparative experiment with multi-objective ant colony optimization (MOACO) algorithm. “Conclusion” makes a conclusion and summarizes future work. The primary processes of this study are shown in Fig. 1.

## Materials and Methods

For convenience, “Problem Definition and Notation” provides the problem definition, related notations, and assumptions, “Mathematical Model” proposes a mathematical model to solve the HVRP-TTW.

### Problem definition and notation

Logically, the model of HVRP-TTW under high temperature is constructed in undirected complete graph $G({V_m},E)$, where ${V_m} = V \cup m$ represents a node set; $V = \left\{ {1,2,...,n} \right\}$ represents a set of customer nodes, whereas $m$ represents the depot in the port chemical park. The arc set $E = \left\{ {(i,j)\left| {i,j \in {V_m},i \ne j} \right.} \right\}$ represents routes between customer nodes. In high-temperature weather, vehicle routes begin at the shared depot and transport hazmat to customer nodes. Each arc $(i,j) \in E$ has a non-negative travel time ${t_{ij}}$, each customer node $i \in V$ has positive integer demand ${q_i}$, and each node has a service time window $(t_e^i,{\rm }t_l^i)$. For convenience, the notation description and decision-making variables are shown in Tables 1 and 2, respectively. Moreover, the following assumptions are given.

(1) All vehicles run or stop in a closed-loop circuit.

(2) There is no limit on the number and destination of shared IBCs shipments.

(3) Available vehicle numbers is sufficient to participate for task in the shared depot.

(4) The speed of vehicles is fixed as a constant.

(5) The temperature, location and customer demand are known at each node.

(6) The maximum loading capacity of vehicles meets the needs of any customer node.

(7) All customer nodes are serviced by the vehicle only once.

(8) The power of each vehicle distance is sufficient to complete its distributed task.

(9) Road conditions are not in the scope of consideration.

(10) The customer's service time window is allowed to expand within a certain range.

**Table 1:** Notation description.

Notation	Description
$m$	Shared port chemical park distribution center
$V$	Set of customer nodes, $V = \left\{ {1,2,...,n} \right\}$
$K$	Set of transport vehicles, $K = \left\{ {1,2,...,k} \right\}$
${V_m}$	Set of customer nodes and shared depot, ${V_m} = V \cup \left\{ m \right\}$
$Q$	Rated load capacity of hazmat vehicle
$a$	Distance travelled per unit weight of vehicle cost
$b$	Cost per vehicle distance traveled
${q_i}$	Customer node $i$ demand, $i \in V$
${q_{ij}}$	The deadweight capacity of the vehicle as it travels from $i$ to $j$, $i \ne j$, $i,j \in V$
${d_{ij}}$	The distance the vehicle travels from $i$ to $j$, $i \ne j$, $i,j \in {V_m}$
${c_1}$	Vehicle fixed cost
${c_2}$	Time window penalties cost
$\alpha$	Temperature class, $\alpha \in \left\{ {I,{\rm \; }II,{\rm \; }III} \right\}$
${t_{ik}}$	The time when the vehicle $k$ arrives at the customer node $i$
$t_e^i$	Minimum time window of node $i$
$t_l^i$	Maximum time window of node $i$
$t_{ei}^\alpha$	The minimum value of the time window specified by the customer node $i$ of the $\alpha$ temperature class
$t_{li}^\alpha$	The maximum value of the time window specified by the customer node $i$ of the $\alpha$ temperature class
$T_{Ei}^\alpha$	The minimum value of the transit time window acceptable to the customer node $i$ of $\alpha$ temperature class
$T_{Li}^\alpha$	The maximum value of the transit time window acceptable to the customer node $i$ of $\alpha$ temperature class
${p^\alpha }$	Time window penalty factor in $\alpha$ temperature class
$\chi$	Penalty factor for exceeding the acceptable time window
$\lambda$	The radius of the affected area at the time of the accident
${\rho _{ij}}$	The population density on the route from $i$ to $j$, $i \ne j$
${h_{ij}}$	Probability of hazmat road transportation accidents on the route from $i$ to $j$, $i \ne j$
$\theta$	Risk factor of hazmat
${\Psi _{ijk}}$	Temperature of the vehicle $k$ as it travels from $i$ to $j$, $i \ne j$
$\bar \Psi$	Temperature threshold
$\bar R$	Risk threshold

**Table 2:** Decision variables used in the model.

Notation	Description
$x_{ij}^k$	Binary variable - 1 if an arc is traveled by a vehicle, 0 otherwise
$x_{mj}^k$	Binary variable - 1 if the vehicle departs from the distribution center, 0 otherwise
${\vartheta _{ik}}$	Binary variable - 1 if the vehicle arrived within an acceptable time frame, 0 otherwise
${\xi _{ik}}$	Binary variable - 1 if the vehicle arrived within an unacceptable time frame, 0 otherwise

### Mathematical model

According to the introduced assumptions, a bi-objective MNLIP model is proposed to solve the HVRP-TTW under high temperature. Concrete analysis of this model is as follows.

(1) Transportation cost of hazmat

① Load driving and fixed costs

In the hazmat transportation process, load driving is the critical factor affecting fuel consumption and transportation cost. According to the research of Xi, Tao & Brooks (2023), the load driving and fixed cost of the hazmat vehicle are obtained as

(1) $${C_1} = \sum\limits_{i = 1}^n {\sum\limits_{j = 1}^n {\sum\limits_{k \in K} {x_{_{ij}}^k{d_{ij}}(a{q_{ij}} + b)} } } + {c_1}\sum\limits_{j = 1}^n {\sum\limits_{k \in K} {x_{mj}^k} } .$$

② Temperature-based time window penalty costs

According to the research of Shen & Wei (2020), temperature variation in summer affects regular hazmat transportation and increases accident risk, including explosions, combustion and leakage. Based on relevant research and surveys from industry professionals, this study establishes temperature thresholds to assess high-temperature conditions. By considering real-world scenarios where daily temperature levels fluctuate and are not constant, even during hot summer days, the study defines temperature levels and proposes a method to adjust customer service time windows accordingly. Set the temperature level $\alpha \in \{ I,II,III\}$ to correspond to different temperatures in high-temperature weather. The looseness of time windows is dynamically adjusted according to the current temperature level during vehicle transportation. Specifically, the extension width of service time at customer nodes is largest at temperature level I, followed by level II, with no change at level III. It is clear that the width of the time window shows the increasing trend during the high temperature weather. In order to reduce the long period of high temperature operation in the current period, a mixed time window is established and a novel temperature-based time window penalty function is proposed as

(2) $$P_{ik}^\alpha \left( {{t_{ik}}} \right) = \left\{ \matrix{0{\rm ,}t_{ei}^\alpha \le {t_{ik}} \le t_{li}^\alpha ; \hfill \cr {p^\alpha }\left( {t_{ei}^\alpha - {t_{ik}}} \right){\rm },T_{Ei}^\alpha \le {t_{ik}} \;\lt\; t_{ei}^\alpha ; \hfill \cr {p^\alpha }\left( {{t_{ik}} - t_{li}^\alpha } \right),t_{li}^\alpha \;\lt\; {t_{ik}} \le T_{Li}^\alpha ; \hfill \cr \chi {\rm ,}T_{Li}^\alpha \;\lt\; {t_{ik}}{\rm }\;or\;{\rm }T_{Ei}^\alpha \;\gt\; {t_{ik}}. \hfill} \right.$$

Then, temperature-based time window cost is obtained as

(3) $${C_2} = {c_2}\left( {\sum\limits_{\alpha = I}^{III} {{p^\alpha } \cdot \sum\limits_{i = 1}^n {\sum\limits_{k \in K} {{\vartheta _{ik}}\left( {(t_{ei}^\alpha - {t_{ik}} \wedge 0) + ({t_{ik}} - t_{li}^\alpha \wedge 0)} \right)} } } } \right) + {c_2}\sum\limits_{i = 1}^n {\sum\limits_{k \in K} {{\xi _{ik}}\chi } } .$$

By using Eqs. (1) to (3), cost function ${f_1}$ of hazmat transportation is obtained as

(4) $$\eqalign{{f_1} = & \;{C_1} + {C_2} \cr =& \sum\limits_{i = 1}^n {\sum\limits_{j = 1}^n {\sum\limits_{k \in K} {x_{_{ij}}^k{d_{ij}}(a{q_{ij}} + b)} } } + {c_1}\sum\limits_{j = 1}^n {\sum\limits_{k \in K} {x_{mj}^k} } \cr &+\; {c_2}\left( {\sum\limits_{\alpha = I}^{III} {{p^\alpha } \cdot \sum\limits_{i = 1}^n {\sum\limits_{k \in K} {{\vartheta _{ik}}\left( {(t_{ei}^\alpha - {t_{ik}} \wedge 0) + ({t_{ik}} - t_{li}^\alpha \wedge 0)} \right)} } } } \right) + {c_2}\sum\limits_{i = 1}^n {\sum\limits_{k \in K} {{\xi _{ik}}\chi } } .}$$

(2) Transportation risk of hazmat vehicle

**Figure 2:**
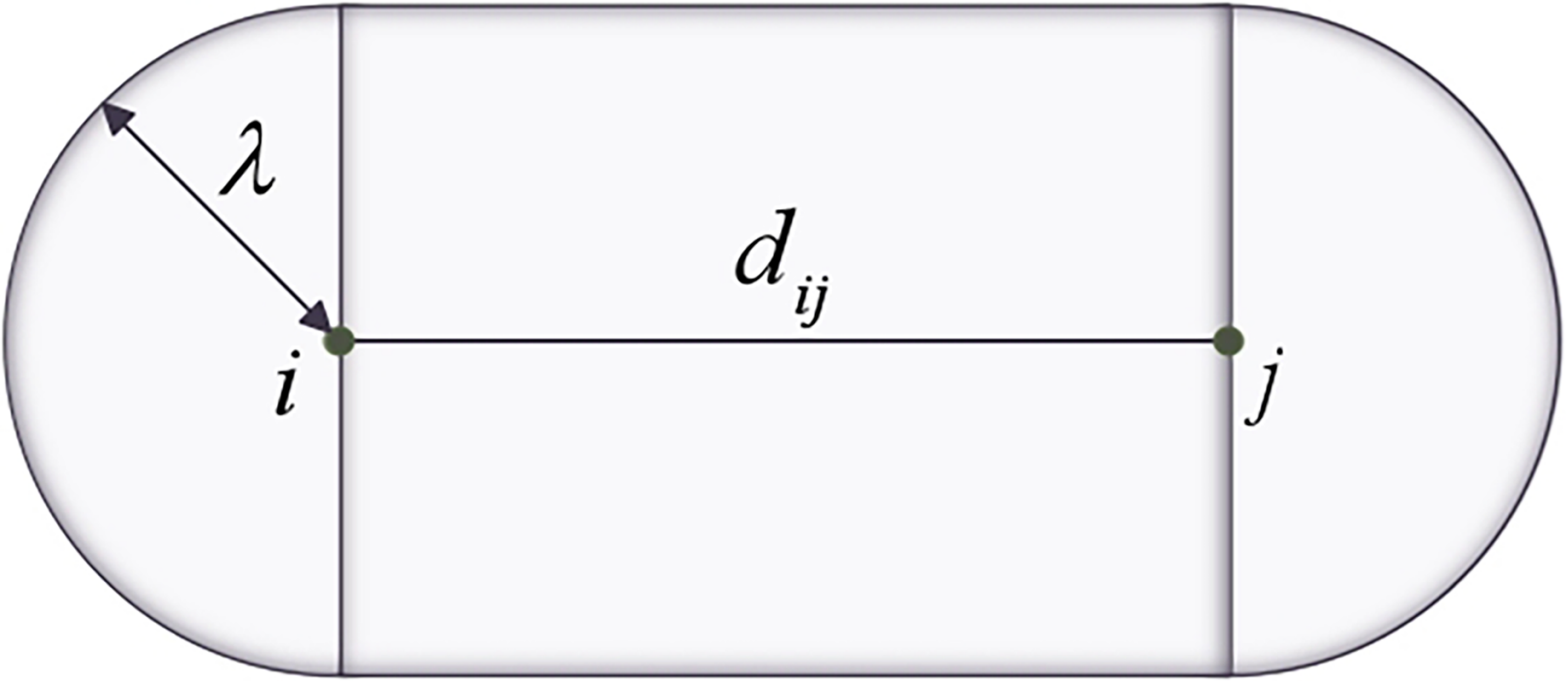
Shape of the affected area around the route segment.

The value of transportation risk is calculated by multiplying the accident probability by the total number of impacted people, according to the conventional risk assessment model. The area of transportation accidents was mainly estimated by casualties (Xing et al., 2020; Huang et al., 2020), and a risk cost function of hazmat vehicle transportation was established with casualty. In the cost function, the casualty index is defined by the population density and the accident extent. Vehicles which move along the transportation route are regarded as a point risk source on the accident point. The shape of affected route segments is shown in Fig. 2, and area ${S_{ij}}$ of affected routes is obtained as

(5) $${S_{ij}} = 2\pi \lambda {d_{ij}} + \pi {\lambda ^2}.$$

For convenience, exposed population $po{p_{ij}}$ is denoted as

(6) $$po{p_{ij}} = {\rho _{ij}} \cdot {S_{ij}}.$$

Meanwhile, the risk of transporting hazmat also increases with the weight of the carried, *i.e*., there is a linear relationship between accident consequences and load capacity (Holeczek, 2021). To quantify the impact of temperature variance on the risk function, temperature threshold is proposed with respect to the hot-weather condition. Specifically, the transportation risk on a given route is defined as

(7) $${R_{ij}} = {h_{ij}}po{p_{ij}}\theta \exp \left( {\displaystyle{{{\psi _{ijk}} - \bar \psi } \over {\bar \psi }}} \right)\displaystyle{{Q - {q_i}} \over Q}.$$

By using Eqs. (5) to (7), the total risk function of hazmat vehicle transportation ${f_2}$ is obtained as

(8) $${f_2} = \sum\limits_{i = 1}^n {\sum\limits_{j = 1}^n {\sum\limits_{k \in K} {{h_{ij}}{\rho _{ij}}\theta \exp \left( {\displaystyle{{{\psi _{ijk}} - \bar \psi } \over {\bar \psi }}} \right)\displaystyle{{Q - {q_i}} \over Q}\left( {2\pi \lambda {d_{ij}} + \pi {\lambda ^2}} \right)} } } .$$

Then, based on the aforementioned derivation process, the bi-objective MNLIP model is obtained as

(9) $$\eqalign{\min {\rm }{f_1} = \; & \sum\limits_{i = 1}^n {\sum\limits_{j = 1}^n {\sum\limits_{k \in K} {x_{_{ij}}^k{d_{ij}}(a{q_{ij}} + b)} } } + {c_1}\sum\limits_{j = 1}^n {\sum\limits_{k \in K} {x_{mj}^k} } \cr &+ {c_2}\left( {\sum\limits_{\alpha = I}^{III} {{p^\alpha } \cdot \sum\limits_{i = 1}^n {\sum\limits_{k \in K} {{\vartheta _{ik}}\left( {(t_{ei}^\alpha - {t_{ik}} \wedge 0) + ({t_{ik}} - t_{li}^\alpha \wedge 0)} \right)} } } } \right) + {c_2}\sum\limits_{i = 1}^n {\sum\limits_{k \in K} {{\xi _{ik}}\chi } } }$$

and

(10) $$\min {\rm }{f_2} = \sum\limits_{i = 1}^n {\sum\limits_{j = 1}^n {\sum\limits_{k \in K} {{h_{ij}}{\rho _{ij}}\theta \exp \left( {\displaystyle{{{\psi _{ijk}} - \bar \psi } \over {\bar \psi }}} \right)\displaystyle{{Q - {q_i}} \over Q}\left( {2\pi \lambda {d_{ij}} + \pi {\lambda ^2}} \right)} } } ,$$

which follows that

(11) $$\sum\limits_{i \in {V_m}} {\sum\limits_{j \in {V_m}} {x_{ij}^k{q_i} \le Q} } ,\;{\rm }\forall k \in K,$$

(12) $$\sum\limits_{i \in {V_m}} {\sum\limits_{k \in K} {x_{ij}^k = 1} } ,\;{\rm }\forall j \in {V_m},$$

(13) $$\sum\limits_{k \in K} {\sum\limits_{j \in {V_m}} {x_{ij}^k} } - \sum\limits_{k \in K} {\sum\limits_{j \in {V_m}} {x_{ji}^k} } = 0,\;{\rm }i \in {V_m},$$

(14) $${q_i} \le Q,\;{\rm }\forall i \in V,$$

(15) $$t_e^{^{III}} \le t_e^{^{II}} \le t_e^{^I},t_l^{^I} \le t_l^{^{II}} \le t_l^{^{III}},T_E^{^{III}} \le T_E^{^{II}} \le T_E^{^I},T_L^{^{III}} \le T_L^{^{II}} \le T_L^{^I},$$

(16) $$f_2^k \le \bar R,\;{\rm }\forall k \in K,$$

(17) $$x_{ij}^k \in \left\{ {0,1} \right\},x_{mj}^k \in \left\{ {0,1} \right\},{\xi _{ik}} \in \left\{ {0,1} \right\},{\vartheta _{ik}} \in \left\{ {0,1} \right\}.$$

Specifically, Eqs. (9) and (10) represent the bi-objective function of hazmat transportation, respectively. Equation (11) represents the vehicle load limit where the total weight of any vehicle $k$ is less than the rated load of vehicles. Equation (12) represents the delivery service on behalf of each yard and only accepts it once. Equation (13) represents that all hazmat vehicles start from and return to the shared depot after completing delivery service. Equation (14) represents customer nodes demand limit. The demand of the node is less than the maximum payload of the vehicle. Equation (15) represents the constraint of time windows at different temperature levels, *i.e*., the looseness of time windows is positively correlated with the temperature class. Equation (16) represents the distribution route risk constraint of a certain vehicle. Equation (17) represents the binary variables.

## Algorithm and model solution procedure

This section introduces a novel ALNS algorithm to address the proposed problem, the basic process of which consists of destruction, repair, and adaptive mechanisms. For convenience, the main content of the algorithm is given in “Adaptive Large Neighborhood Search”, and the solving process of the model is given in “Model Solution Procedure”.

### Adaptive large neighborhood search

The ALNS mainly selects efficient destroying and repairing neighborhood structures (operators) by using an adaptive strategy in the iterative process to obtain high-quality solutions. For convenience, an improved ALNS algorithm uses novel heuristic operators to expand the neighborhood search range and employs a phased operator weight and score update method to avoid falling into local optimal solution. The pseudo-code of an ALNS algorithm is presented in Table 3.

**Table 3:** The pseudo-code of the proposed ALNS algorithm.

ALGORITHM ALNS.
1: **Input:** the initial solution ${s_0}$;
2: Initialization parameters;
3: ${s_1} \leftarrow {s_0}$; ${s^*} \leftarrow {s_0}$;
4: **While** $seg \le max\_seg$ **do**
5: **While** $iter \le seg$ **do**
6: $s_1^{\prime} \leftarrow$ Select a destroy operator to remove nodes from ${s_1}$;
7: ${s_2} \leftarrow$ Select a repair operator to insert nodes into $s_1^{\prime}$;
8: **If** $F\left( {{s_2}} \right) \;\lt\; F\left( {{s^*}} \right)$ **then**
9: ${s_1} \leftarrow {s_2}$; ${s^*} \leftarrow {s_2}$; The scores of used operators are added by ${{\rm \sigma }_1}$;
10: **Else if** $F\left( {{s_2}} \right) \;\lt\; F\left( {{s_1}} \right)$ **then**
11: ${s_1} \leftarrow {s_2}$; The scores of used operators are added by ${{\rm \sigma }_2}$;
12: **Else if** meet the Metropolis criterion, **then**
13: ${s_1} \leftarrow {s_2}$; The scores of used operators are added by ${{\rm \sigma }_3}$;
14: **Else**
15: The scores of used operators are added by ${{\rm \sigma }_4}$;
16: **End if**
17: $iter \leftarrow iter + 1$;
18: **End while**
19: Update the weights and scores of all operators;
20: $seg \leftarrow seg + 1$;
21: **End while**
22: **Output:** the best solution ${s^*}$.

#### Generate initial solution

Initial solution within constraints is generated using a greedy algorithm. Specifically, vehicle starts from the depot and calculates nodes closest to its current location. Then, determine whether the node meets the load limit. If satisfied, drive to the node; if not satisfied, return to the depot. Such a closed loop is a vehicle route, and a new vehicle departs from the depot. Until all nodes are accessed, all vehicle routes containing all customer nodes are an initial feasible solution.

#### Destroy operators

The destroy operator removes partial nodes from the current solution and generates an intermediate solution ${{s}^{\prime}_1}$ after the destruction. In each iteration, an operator is selected to remove ${d_n}$ nodes from the solution which are added to list ${L_d}$. Among them, ${d_n}$ is given by ${d_n} = \mu \cdot n$, where $\mu$ indicates the degree of destruction. In this subsection, four destroy operators are detailed, named random removal, worst removal, Shaw removal, and random importance removal. The first three are classical destroy operators (Ropke & Pisinger, 2006; Shaw, 1997), and the last one is a novel destroy operator proposed by combining the importance degree of customer nodes.

**Random removal**: The customer nodes in the vehicle route are randomly removed and then these nodes are stored in list ${L_d}$.

**Worst removal**: Remove customer nodes from the current vehicle routes to achieve the maximum saving value. The saving value represents the cost difference before and after the removal of customer nodes from the current solution.

**Shaw removal**: Select a node randomly and remove the node of the highest similarity to it. Repeat this process until enough nodes have been removed. The similarity is relevant to distance, time window and demand. The similarity $R(i,j)$ is as follows

(18) $$R\left( {i,j} \right) = {\delta _1}\displaystyle{{{d_{ij}}} \over {\mathop {\max }\limits_{i,j \in V} {d_{ij}}}} + {\delta _2}\left( {\displaystyle{{\left| {t_e^i - t_e^j} \right|} \over {\mathop {\max }\limits_{i,j \in V} \left| {t_e^i - t_e^j} \right|}} + \displaystyle{{\left| {t_l^i - t_l^j} \right|} \over {\mathop {\max }\limits_{i,j \in V} \left| {t_l^i - t_l^j} \right|}}} \right) + {\delta _3}\displaystyle{{\left| {{q_i} - {q_j}} \right|} \over {\mathop {\max }\limits_{i,j \in V} \left| {{q_i} - {q_j}} \right|}},$$

where ${\delta _1}$, ${\delta _2}$, and ${\delta _3}$ are the weights.

**Random importance removal**: Calculate the importance of all customer nodes. According to their importance values, nodes are sorted and removed in descending order. Then, the relevance value ${R_i}$ is obtained as

(19) $${R_i} = \displaystyle{{{d_{mi}}} \over {\mathop {\max }\limits_{i \in V} {d_{mi}}}} + \displaystyle{{t_l^i - t_e^i} \over {\mathop {\max }\limits_{i \in V} \left( {t_l^i - t_e^i} \right)}} + \displaystyle{{{q_i}} \over {\mathop {\max }\limits_{i \in V} {q_i}}}.$$

#### Repair operators

In this subsection, two repair operators (greedy insertion and regret insertion) are selected from classical operators, and a novel operator is introduced based on the importance of customers. The function of repair operators is to reinsert removed nodes into ${{s}^{\prime}_1}$, provided that constraints are met. When calculating the function value change after node insertion, other nodes without position change are ignored. All repair operators run until there are no customer nodes in list ${L_d}$. Consequently, ${{s}^{\prime}_1}$ is updated to a new feasible solution. A detailed introduction to the repair operators is provided as follows.

**Greedy insertion**: Select a customer node from list ${L_d}$ and insert into ${{s}^{\prime}_1}$. Specifically, the cost change of the solution is calculated for inserting a node at each potential insertion position. Once the node is inserted into the vehicle route position with the smallest cost change, it is removed from the list. Until the list is empty, repeat the previous process.

**Regret insertion**: The regret value reflects the cost difference between placing a customer node in the optimal position and a sub-optimal one. For each node and each potential insertion position, calculate the cost difference resulting from inserting the node. Then, the optimal $\Delta f_i^1$ and sub-optimal $\Delta f_i^2$ differences for node $i$ are obtained. The position with the smallest difference is the optimal, while the second smallest is the sub-optimal. Calculate the regret values for all nodes based on ${\Delta_i} = \mathop {\max }\limits_{i \in {L_d}} \left\{ {\Delta f_i^2 - \Delta f_i^1} \right\}$, and select a node with the highest regret value. The node is inserted into the corresponding optimal position and removed from list ${L_d}$. Update the regret values of the nodes and reinsert them until no nodes are left in the list.

**Greedy importance insertion**: According to Eq. (19), the importance values of customer nodes in the list are calculated, and select a node with the highest importance. Then, calculate the objective function change value for this node at all potential insertion positions. Insert the node where it causes the least change and update the route accordingly. Continue selecting nodes and inserting them based on the importance value until the list is empty.

### Model solution procedure

Based on the main algorithm description, the solution procedure for the HVRP-TTW model is as follows.

**Step 1** Initialization. A greedy algorithm generates the initial solution ${s_0}$ while ensuring the vehicle load constraint is satisfied. Global optimal solution ${s^ * }$ and current solution ${s_1}$ are assigned as ${s_0}$. Then all parameters are initialized.

**Step 2** Selection of operators. A probabilistic roulette wheel selection mechanism is used to select a heuristic operator. The operator weights are updated after one segment, where one segment represents $\varphi$ iterations. In segment $seg$, operator $o$ with weight $\omega _o^{seg}$ is chosen with probability $p_o^{seg}$ where $p_o^{seg} = \displaystyle{{\omega _o^{seg}} \over {\sum\nolimits_{i = 1}^r {\omega _i^{seg}} }}$, and $r$ represents the number of operators. Specifically, the initial value of all operator weights is 1.

**Step 3** Generate a new solution. The new solution ${s_2}$ is generated after the destroy and repair operations of ${s_1}$ using the selected operators.

**Step 4** Update the global optimal solution. According to Eqs. (9) and (10), the bi-objective values of ${s^ * }$, ${s_1}$ and ${s_2}$ are calculated. If the bi-objective values of ${s_2}$ are better than ${s^ * }$, *i.e*., $F({s_2}) \le F({s^ * })$, ${s_1}$ and ${s^ * }$ are updated to ${s_2}$. $F( \cdot )$ is the fitness value of solution, $F( \cdot ) = \displaystyle{{{f_1}( \cdot ) + {f_2}( \cdot )} \over 2}$. Then, the scores of the selected operators are added by ${\sigma _1}$. Otherwise, go to Step 5.

**Step 5** Update the current solution. If $F({s_2}) \le F({s_1})$, ${s_1}$ is updated to ${s_2}$. The scores of the selected operators are added by ${\sigma _2}$. Otherwise, go to Step 6.

**Step 6** Update with probability. If $F({s_2}) > F({s_1})$, according to the Metropolis criterion of simulated annealing, ${s_2}$ is accepted as ${s_1}$, with the acceptance probability ${e^{ - {{\left( {F\left( {{s_2}} \right) - F\left( {{s_1}} \right)} \right)}\mathord /T}}}$ obtained by borrowing from Mohri et al. (2020). $T$ represents the temperature, $T = \partial T$ after each iteration, and $\partial$ denotes the annealing rate. The scores of selected operators are added to ${\sigma _3}$ if the ${s_2}$ is accepted and ${\sigma _4}$ if no updates are accepted.

**Step 7** Update operator weights. After each segment, the operator weights $\omega _o^{seg}$ are updated. $\omega _o^{seg + 1} = (1 - \eta )\omega _o^{seg} + \displaystyle{{\eta g_o^{seg + 1}} \over {v_o^{seg + 1}}}$ is the weight update formula, where $\eta$ represents the react factor. $g_o^{seg + 1}$ represents the total scores obtained by operator $i$ in segment $seg + 1$, and $v_o^{seg + 1}$ denotes the number of times operator $i$ is selected in segment $seg + 1$.

**Step 8** Output the result. The iteration is stopped until the number of iterations is completed. Then, output ${s^ * }$.

## Results and discussion

For convenience, this section contains four subsections, which are described below. “Data Set” presents information on the dataset and the relevant parameter settings for the ALNS algorithm. “Results and Analysis” gives the solution steps and running results of the mathematical model in the algorithm. “Comparative analysis” provides a comparative analysis to evaluate the effectiveness of the ALNS algorithm. “Discussion” discusses the model in relation to existing research and analyzes its advantages and disadvantages. The ALNS algorithm was implemented using PyCharm, and experiments were run on an Intel core i5-13500H 2.60GHz computer with Windows 11.

### Data set

To evaluate the performance of the ALNS algorithm, this study utilized 18 VRPTW instances of various scales proposed by Solomon (1987). The source data is available at Solomon Benchmark (2008). It is well known that the Solomon dataset contains instances with six different classes of features. Two instances are selected from each class, and each instance is divided into three scales of customer nodes for testing. Those instances with 25, 50, and 100 customer nodes, denoted as C101.25, C101.50, and C101.100 respectively. To adapt the scenarios in this study, the dataset is modified according to the Solomon VRPTW instance. The main modifications are as follows.

(1) The service times of customer nodes are removed from the original Solomon instances.

(2) The maximum service time of the warehouse is divided equally into 24 segments, which are considered as the cut-off point of 24 h in descending order.

(3) As the temperature varies, the service time windows are adjusted during different periods, and the adjustment amount is linked to the temperature category.

**Figure 3:**
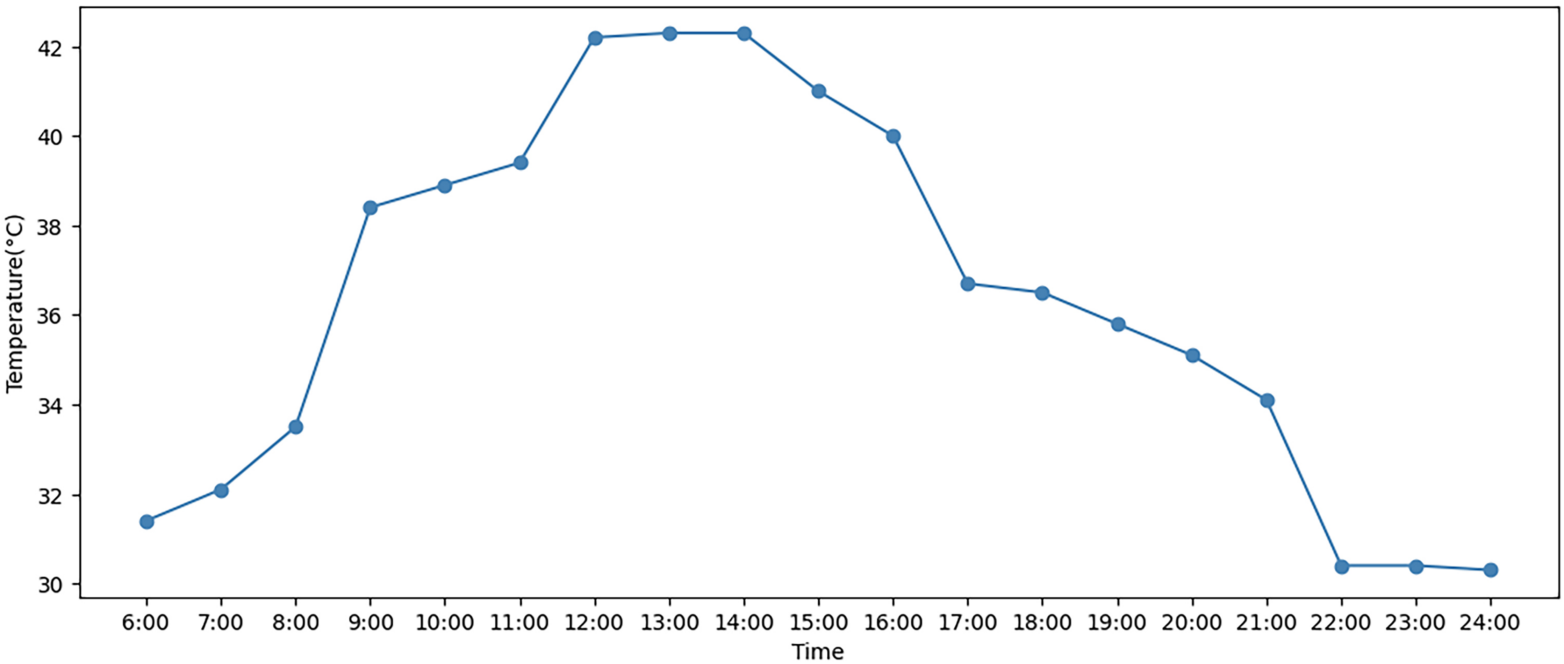
Hourly temperature data in the summer of July 2023.

In addition, the hourly temperature change for a day in July 2023 is obtained from the weather forecast (Hersbach et al., 2018). Figure 3 shows the temperature data. Table 4 presents the values of all parameters utilized in the ALNS algorithm. The values of some parameters are derived from previous studies, and the values of other parameters are determined in combination with the actual situation (Ropke & Pisinger, 2006; Lin et al., 2023).

**Table 4:** The parameters used in the ALNS algorithm.

Description	Parameter	Numerical value
Algorithm parameters	$N$	1,000
	$\phi$	10
	$\mu$	0.3
	$\sigma_1, \sigma_2, \sigma_3, \sigma_4$	5, 3, 1, 0
	$\delta_1, \delta_2, \delta_3$	9, 6, 2
	$\eta$	0.5
	$\partial$	0.99975
Model parameters	$a, b$	0.1, 1
	$c_1, c_2$	500, 30
	$p^{I}, p^{II}, p^{III}$	0.4, 0.3, 0.2
	$h$	5.83 × 10^-7^
	$\lambda$	5
	$\theta$	1
	$\bar\psi$	35
	$\bar{R}$	0.1

### Results and analysis

**Figure 4:**
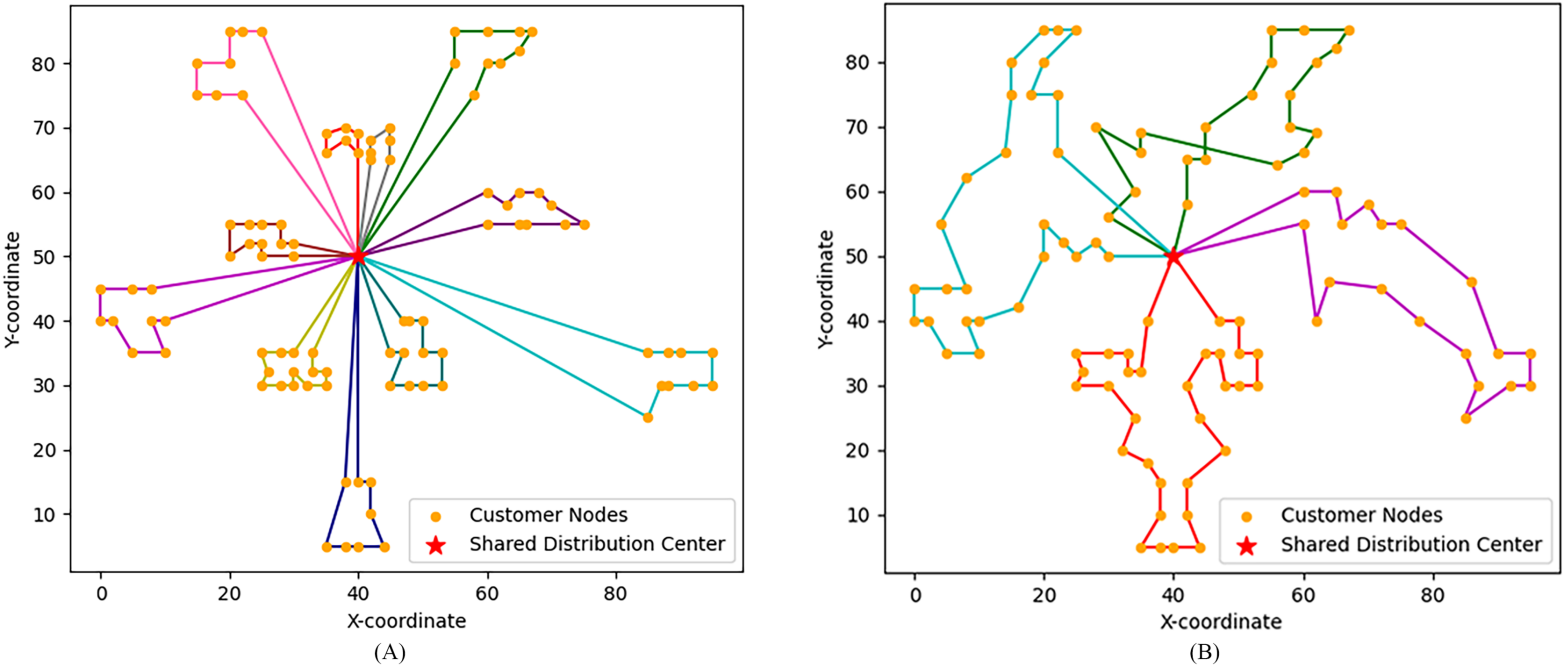
Vehicle transport routes for two instances. (A) C101.100 vehicle routes. (B) C201.100 vehicle routes.

**Figure 5:**
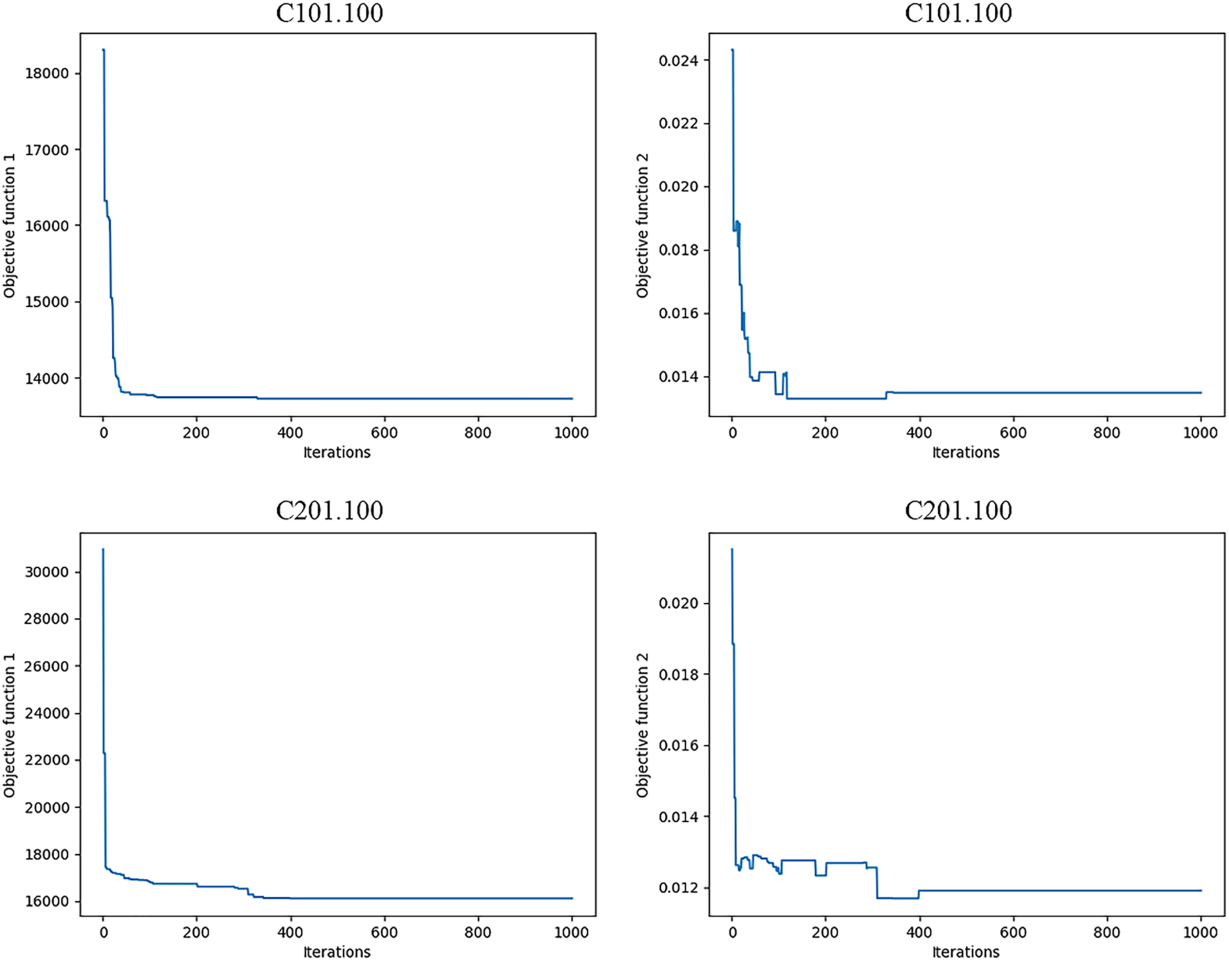
Bi-objective value iteration procedures for two instances.

Obviously, the characteristics of each class of examples are different, mainly reflected in the geographical location distribution of customer nodes and other aspects. The customer locations of class C examples are reflected in the form of clusters, class R is completely randomly generated, and class RC combines the characteristics of the first two types, with both cluster distribution and random distribution. Therefore, different types of instances are selected in this subsection and the algorithm steps described in ‘Model solution procedure’ are used to solve HVRP-TTW. Table 5 shows the specific run results of solving HVRP-TTW in different instances, each of which is run ten times in the ALNS algorithm. For convenience, Fig. 4 shows the optimal vehicle transportation routes for the two instances C101.100 and C201.100, and Fig. 5 shows how the bi-objective function values change during the iterations for these two instances.

**Table 5:** Results of the HVRP-TTW instances.

Instance	Vehicles	BS		AS		Time
		*f*_1_	*f*_2_	*f*_1_	*f*_2_	
C101.25	3	3,211.01	0.00289	3,215.71	0.00289	3.45
C101.50	5	5,927.12	0.00532	5,927.12	0.00532	15.37
C101.100	11	13,282.15	0.0125	13,496.15	0.01302	148.48
C201.25	3	3,954.07	0.00382	4,188.86	0.00448	5.02
C201.50	3	7,879.64	0.00808	8,361.16	0.00843	46.85
C201.100	4	16,114.39	0.01191	16,329.65	0.01225	363.97
R101.25	8	5,797.14	0.01154	5,850.14	0.01175	6.41
R101.50	13	10,117.51	0.01926	10,204.06	0.01964	35.06
R101.100	23	17,902.01	0.03343	18,476.6	0.03381	238.4
R201.25	2	4,735.15	0.00925	4,803.63	0.00927	8.12
R201.50	4	8,588.05	0.01554	10,062.62	0.01605	65.42
R201.100	6	15,573.64	0.0257	16,621.55	0.0256	451.06
RC101.25	3	4,685.03	0.00445	4,690.2	0.00446	3.79
RC101.50	9	9,906.05	0.01425	10,014.91	0.01453	27.58
RC101.100	18	17,725.15	0.02878	18,083.6	0.02963	267.13
RC201.25	2	5,612.17	0.0067	5,622.09	0.00676	6.67
RC201.50	4	13,252.6	0.017	13,653.34	0.0143	53.85
RC201.100	6	19,972.19	0.02328	21,456	0.02639	337.44

**Note:**

BS is the best solution and AS is the average solution.

In Table 5, the first column denotes the instance label, the second column indicates the number of utilized vehicles, and the subsequent three columns display the bi-objective function values of both best solution (BS) and average solution (AS) in the runtime results, along with the average running time.

In Fig. 4, aclosed loop connecting customer points with the same colour is the transportation route of one vehicle, that is, C101.100 uses 11 vehicles and C201.100 uses four vehicles. In Fig. 5, analyzing the bi-objective value iteration for two instances simultaneously reveals that fluctuations in the value of the second objective function are more pronounced during initial iterations. This is due to its dependency on the first objective function. This implies a scenario where one objective value decreases while another increases. Simultaneously, as part of iterative process, algorithm escapes from local optimal solutions which aids in identifying global optimal solution prior to conclusion of iterations. The bi-objective function values remain notably stable for at least 200 iterations before completion of runtime. Consequently, a feasible solution at this stage is considered as an optimal solution, and its corresponding path is deemed as an optimal path.

To further investigate the influence of temperature-adjusted time window widths, we conducted a sensitivity analysis on the HVRP-TTW model. Based on the results from the model above, parameters related to time window adjustments for two temperature levels were modified. Specifically, within the model, the service time window expands by 1x on each side at temperature level III, and by 0.5x on each side at level II. The tests are conducted for three size instances of instance C101.

**Figure 6:**
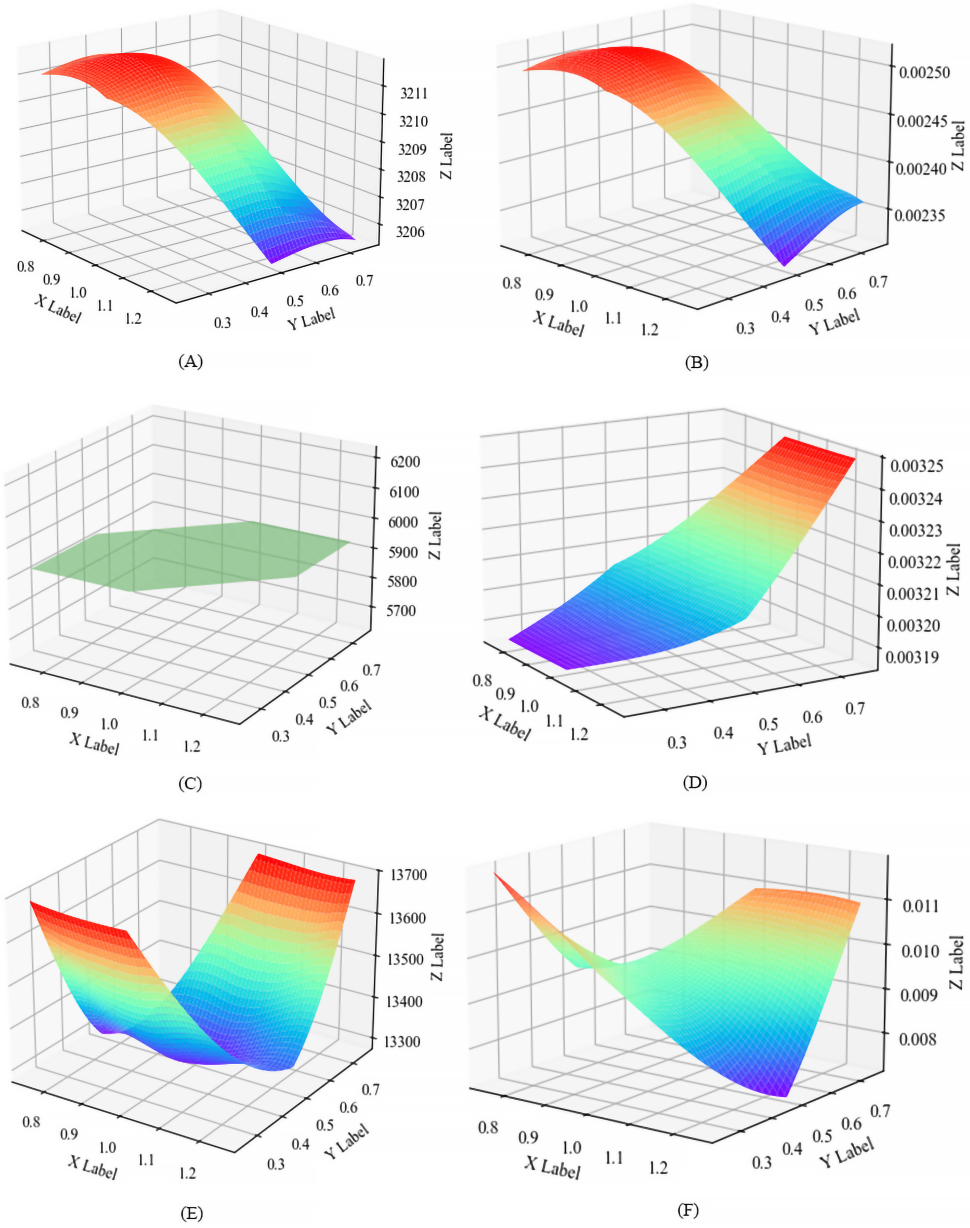
Results produced in different parameter settings. (A) The parameter set to the cost value of (1.25, 0.75). (B) Set the parameter to the risk value of (0.75, 0.25). (C) Set the parameter to the cost value of (1.25, 0.5). (D) The parameter to the risk value of (0.75, 0.5). (E) Set the parameter to the cost value of (1, 0.75). (F) The parameter set to the risk value of (1, 0.25).

The width of the customer service time window is a key parameter that determines the sequence of nodes for transportation vehicles and influences total cost and total risk. By integrating temperature levels, we tested the impact of two parameters on the results, setting different expansion ratios, specifically (1.25, 0.75), (0.75, 0.25), (1.25, 0.5), (0.75, 0.5), (1, 0.75) and (1, 0.25), as illustrated in Fig. 6. In terms of node scale, the results indicate that transportation cost and risk decrease with increasing time window expansion ratios, which shows minimal variation in small-scale instances. Besides, the setting temperature level in the large ratio leads to better solution while the ratio at level II is not necessary for the large-scale instances. Therefore, we can conclude that the greater the adjustment of the customer service time window under high-temperature conditions, the better the risk reduction and the lower the total operational costs.

### Comparative analysis

To demonstrate the effectiveness of the ALNS algorithm, 18 instances of Solomon VRPTW are first solved and compared with known studies. Additionally, ALNS algorithm and MOACO algorithm are used to solve HVRP-TTW. Each instance is run 10 times, and the performance of the two algorithms is compared according to the running time and results. The number of ants in MOACO is 10, the pheromone evaluation is 0.1, and the heuristic factor is 2.

Table 6 shows comparative results of solving VRPTW including the instances, the best-known solutions (BKS), results and deviations of the proposed ALNS algorithm. The objective is to minimize the number of vehicles (NV) and the total distance (TD). NV_a_ and NV_b_ represent the number of vehicles in BKS and the optimal solution obtained by the ALNS algorithm, respectively. BD_a_ denotes the best distance of BKS. TD_b_ and TD_a_ denote the best and average total distances obtained by the ALNS algorithm, respectively. The deviation represents the variance between the optimal distance attained by the ALNS algorithm and the best distance in BKS, *i.e*., $\displaystyle{{{\rm T}{{\rm D}_{\rm b}} - {\rm B}{{\rm D}_{\rm a}}} \over {{\rm B}{{\rm D}_{\rm a}}}} \times 100 \%$. Six cases showed new solutions, and one case had a 0.00% deviation, all highlighted in bold. In the Supplemental Data, Table S1 displays the specific path details of the new solution, including nodes for each path, travel distance and time. Furthermore, the deviation of the other instances is within 7%, demonstrating the robustness of the ALNS algorithm in solving instances of various sizes.

**Table 6:** Results of VRPTW instances in Solomon. The algorithm obtains some new solutions, which have fewer vehicles or lower total distance than BKS, so some deviations are negative (see bold entries in this table).

Instance	BKS		Refs.	ALNS			Deviation
	NV_a_	BD_a_		NV_b_	TD_b_	TD_a_	
C101.25	3	191.3	Kohl et al. (1999)	3	191.81	191.81	0.27%
C101.50	5	362.4	Kohl et al. (1999)	5	363.25	363.25	0.23%
C101.100	10	827.3	Kohl et al. (1999)	10	828.94	828.94	0.20%
C201.25	2	214.7	AUREN (2006)	2	215.54	215.54	0.39%
**C201.50**	3	360.2	AUREN (2006)	3	361.8	475.56	0.44%
				**2**	**444.96**		
**C201.100**	3	591.58	Tan, Chew & Lee (2006)	3	591.56	605.53	**0.00%**
R101.25	8	617.1	Kohl et al. (1999)	8	618.33	634.69	0.20%
R101.50	12	1,044	Kohl et al. (1999)	12	1,050.97	1,059.48	0.67%
R101.100	19	1,648.08	Wu et al. (2024)	20	1,655.59	1,667.22	0.46%
R201.25	4	463.3	AUREN (2006)	3	474.37	474.37	2.39%
**R201.50**	6	800.7	AUREN (2006)	**4**	**817.19**	817.19	2.06%
**R201.100**	5	1,206.42	Tan, Chew & Lee (2006)	**6**	**1,173.26**	1,220.72	**−2.75%**
RC101.25	4	461.1	Kohl et al. (1999)	4	462.16	469.56	0.23%
**RC101.50**	9	957.9	AUREN (2006)	**8**	**946.46**	970.31	**−1.19%**
RC101.100	15	1,619.8	Kohl et al. (1999)	16	1,718.86	1732.14	6.12%
RC201.25	3	360.2	AUREN (2006)	3	361.24	396.77	0.29%
**RC201.50**	5	684.8	AUREN (2006)	**4**	**714.97**	714.97	4.41%
**RC201.100**	6	1,302.81	Lin et al. (2023)	**7**	**1,291.63**	1,339.75	**−0.86%**

**Note:**

BKS is the best-known solutions. The objective is to minimize the number of vehicles (NV) and the total distance (TD). NV_a_ and NV_b_ represent the number of vehicles in BKS and the optimal solution obtained by the ALNS algorithm, respectively. BD_a_ denotes the best distance of BKS. TD_b_ and TD_a_ denote the best and average total distances obtained by the ALNS algorithm, respectively.

Table 7 shows the detailed results of HVRP-TTW instances solved by MOACO algorithm, including the best solution, average solution and average running time of each instance, which is similar to Table 4. It is easy to see that vehicle numbers used in the results obtained by the MOACO algorithm is generally less than that of an ALNS algorithm. However, the optimization result of the ALNS algorithm for the target value is better.

**Table 7:** Results of HVRP-TTW instances by the MOACO algorithm.

Instance	Vehicles	BS		AS		Time
		*f*_1_	*f*_2_	*f*_1_	*f*_2_	
C101.25	3	3,215.65	0.00298	3,295.7	0.00314	226.58
C101.50	5	7,492.8	0.009	7,764.88	0.00903	300.49
C101.100	10	18,485.1	0.02131	20,543.63	0.024	562.83
C201.25	1	9,321.48	0.00394	9,444.99	0.00392	196.43
C201.50	2	30,243.53	0.01253	35,793	0.01402	186.71
C201.100	3	70,789.42	0.02904	82,788.7	0.03037	312.31
R101.25	4	4,471.96	0.00872	4,479.51	0.00864	202.59
R101.50	8	8,889.31	0.01644	8,988.98	0.01657	285.92
R101.100	11	16,883.12	0.02434	17,255.6	0.02206	992.57
R201.25	1	8,314.51	0.00951	8,339.38	0.00962	184.765
R201.50	2	27,400.2	0.02326	28,106.5	0.02233	304.498
R201.100	2	60,895.12	0.02324	64,318.7	0.02416	394.66
RC101.25	3	4,653.02	0.00441	4,782.98	0.00465	343.81
RC101.50	5	9,268.27	0.008	9,404.92	0.00794	521.12
RC101.100	9	18,736.6	0.01843	19,496.4	0.01883	1,123.6
RC201.25	1	12,051.45	0.00715	12,307.37	0.00717	228.65
RC201.50	2	35,941.84	0.02299	39,164.04	0.02191	184.81
RC201.100	6	73,019.88	0.02125	77,970.4	0.0214	467.3

Table 8 shows the comparative information of the above two algorithms, including the number of vehicles used and the percentage difference of the objective function value and running time for the two. Here, the deviation is given as a percentage, *i.e*., $\displaystyle{{{\rm B}{{\rm S}_{{\rm ALNS}}} - {\rm B}{{\rm S}_{{\rm MOACO}}}} \over {{\rm B}{{\rm S}_{{\rm MOACO}}}}} \times 100 \%$. It is obvious that the ALNS algorithm has better convergence effect when solving the instances with customer nodes aggregation, such as the C class and the RC class. Correspondingly, the MOACO algorithm performs better when dealing with the problems with randomly distributed coordinate points. In addition, although the number of vehicles obtained by the MOACO algorithm is more optimal, the ALNS algorithm finds the optimal solution more efficiently in most instances. It is evident that the ALNS algorithm demonstrates flexibility in adjustments and effectiveness convergence when addressing instances with smaller time windows.

**Table 8:** Detailed comparison results of the ALNS algorithm and the MOACO algorithm.

Instance	Vehicles		Deviation		
	MOACO	ALNS	*f*_1_	*f*_2_	Time
C101.25	3	3	−0.14%	−3.02%	−98.48%
C101.50	5	5	−20.90%	−40.89%	−94.89%
C101.100	10	11	−28.15%	−41.34%	−73.62%
C201.25	1	3	−57.58%	−3.05%	−97.44%
C201.50	2	3	−73.95%	−35.51%	−74.91%
C201.100	3	4	−77.24%	−58.99%	16.54%
R101.25	4	8	29.63%	32.34%	−96.84%
R101.50	8	13	13.82%	17.15%	−87.74%
R101.100	11	23	6.03%	37.35%	−75.98%
R201.25	1	2	−43.05%	−2.73%	−95.61%
R201.50	2	4	−68.66%	−33.19%	−78.52%
R201.100	2	6	−74.43%	10.59%	14.29%
RC101.25	3	3	0.69%	0.91%	−98.90%
RC101.50	5	9	6.88%	78.13%	−94.71%
RC101.100	9	18	−5.40%	56.16%	−76.23%
RC201.25	1	2	−53.43%	−6.29%	−97.08%
RC201.50	2	4	−63.13%	−26.05%	−70.86%
RC201.100	6	6	−72.65%	9.55%	−27.79%

### Discussion

These results show that an effective adjustment time window reduces the total transportation cost and risk. In addition, the damage and repair operators are added to the ALNS algorithm, which expands the search range of the neighborhood and provides more possibilities for finding better solutions.

By testing a series of Solomon benchmark instances, this study analyzed the impact of varying the looseness of the service time windows based on temperature levels on the computational results. The results indicate that the performance of the model is related to the characteristics of the test instances. Specifically, the optimization is more effective for clustered distributions and random distributions with fewer than 100 nodes. The improved ALNS algorithm exhibits smaller objective value errors and some negative values when handling these distributed instances, while the errors remain within a reasonable range in other cases. This suggests that the proposed algorithm performs well across various application scenarios. Furthermore, temperature-based time windows effectively reduce transportation costs and risks for customers. Variable time windows offer customers more economically secure options and expand the range for adjusting vehicle transportation routes, thereby enhancing the efficiency of vehicle transportation. This further demonstrates the effectiveness of the proposed model in this study. Therefore, the HVRP-TTW model is suitable for VRP in high-temperature conditions.

In the aspect of practical applications, the implementation of the proposed model is built in the existing logistics framework, which is the emphasis for decision-makers with a critical evaluation of route choice. Concretely, decision-makers begin with a thorough evaluation of logistics strategies to identify where sensitivity to temperature conditions exists. Then, the active cooperation in transportation and production ensures smooth operation of logistics. Thus, model performance should be reviewed regularly based on feedback to make adjustments timely and sustain its effectiveness.

Although the numerical examples have obtained meaningful results, there are still some limitations. When constructing the problem, the model does not consider the influence of dynamic factors such as traffic flow. In the actual transportation environment, there are many risk factors causing accidents, and it is difficult to quantify the subjective situation. Then, the model faces severe challenges when considering the quantification of multiple factors to reduce the risk of decision-making.

## Conclusions

In this study, a kind of HVRP-TTW is introduced to quantitatively analyse the influence of high-temperature weather on hazmat transportation risk. Moreover, a novel optimal model and a corresponding ALNS algorithm have been provided. Specifically, the following conclusion is drawn.

Firstly, this study introduces a novel pattern of hazmat transportation using sharing IBCs, which is inspired by the idea of container sharing. This pattern enhances transportation efficiency through sharing, and effectively lowers transportation costs by facilitating interoperability between various customer nodes.

Secondly, a bi-objective MINLP model for HVRP-TTW is refined, which considers the total cost and risk in high-temperature weather transportation as its objectives. The model integrates temperature-based time windows to address the impact of temperature fluctuations on transportation efficiency and risk.

Thirdly, this study introduces a novel series of destroy and repair operators that extend the neighborhood search range, thereby enabling the ALNS algorithm to explore globally optimal solutions efficiently. Subsequently, the algorithm's efficacy is validated by the reconstructed Solomon benchmark instances. In light of the interests of all parties and the actual decision-making situation, a compromise selection of different optimization objectives is made to meet the constraints of complex problems.

For decision-makers in hazmat transportation, the method studied by adjusting route choice based on the variance in environment conditions, offers a significant managerial implication to improve transportation safety and efficiency. In practice, the probability of accidents caused by temperature changes is unknown, discrete and fuzzy parameter setting may reduce transportation risks. As a further study, it is a choice to consider multi-type hazmat vehicles with fuzzy demands.

## Supplemental Information

10.7717/peerj-cs.2586/supp-1 Supplemental Information 1 New solutions for VRPTW instances in Solomon.

10.7717/peerj-cs.2586/supp-2 Supplemental Information 2 Datasets and code.

## Additional Information and Declarations

### Data Availability

The following information was supplied regarding data availability: The code is available in the Supplemental File. The Solomon benchmark data (with 25, 50, and 100 customer nodes) are available at SINTEF: https://www.sintef.no/projectweb/top/vrptw/solomon-benchmark.
