# Supplementary material for: A novel optimization method for hazardous materials vehicle routing with temperature-based time windows
Source: PeerJ Comput Sci. 2024 Dec 13;10:e2586. doi: 10.7717/peerj-cs.2586 (PMC11784799; doi:10.7717/peerj-cs.2586)
Supplement: Supplemental Information 1 [file peerj-cs-10-2586-s001.docx]

Table S1:

New solutions for VRPTW instances in Solomon.

| **Instance** | **Routes** | **Load** | **Distance** | **Duration** |
| --- | --- | --- | --- | --- |
| C201.50 | [0,5,2,1,49,7,3,4,40,44,46,45,50,47,43,42,41,48,0] | 230 | 249.66 | 2956.83 |
|  | [0,20,22,24,27,30,29,6,32,33,31,35,37,38,39,36,34,28,26,23,18,  19,16,14,12,15,17,13,25,9,11,10,8,21,0] | 630 | 195.3 | 3165.3 |
| R201.50 | [0,5,45,36,47,19,11,7,18,6,8,49,46,48,17,0] | 201 | 244.63 | 846.82 |
|  | [0,27,33,29,12,31,30,9,34,3,50,20,10,32,5,1,0] | 225 | 258.19 | 760.72 |
|  | [0,2,42,15,14,44,16,38,43,37,13,0] | 131 | 104.4 | 711.18 |
|  | [0,28,39,23,21,41,22,40,26,4,25,24,0] | 164 | 173.96 | 882 |
| R201.100 | [0,72,39,67,23,75,73,40,53,87,57,41,22,56,4,54,55,25,24,80,77,0] | 290 | 216.79 | 907.63 |
|  | [0,28,33,65,71,9,51,81,79,78,34,50,3,68,26,0] | 233 | 160.58 | 682.39 |
|  | [0,31,63,11,64,49,46,48,0] | 125 | 128.62 | 810.8 |
|  | [0,5,83,45,82,47,36,19,62,88,7,18,8,84,17,91,100,93,60,89,0] | 239 | 192.66 | 872.11 |
|  | [0,95,92,14,42,15,2,21,12,29,76,30,90,10,20,66,35,32,70,1,0] | 234 | 264.92 | 831.29 |
|  | [0,27,69,52,59,98,61,16,44,38,86,85,99,94,6,96,97,37,43,74,13,58,0] | 337 | 209.69 | 868.06 |
| RC101.50 | [0,42,44,3,1,0] | 50 | 109.76 | 193.08 |
|  | [0,39,36,38,41,40,43,37,35,0] | 180 | 128.81 | 216.25 |
|  | [0,31,29,27,26,32,0] | 90 | 121.37 | 194.08 |
|  | [0,23,21,19,18,48,25,0] | 130 | 112.09 | 223.28 |
|  | [0,11,22,49,20,24,0] | 110 | 119.8 | 195.27 |
|  | [0,5,45,2,7,6,8,46,4,0] | 160 | 107.48 | 188.17 |
|  | [0,14,47,12,15,16,9,10,13,17,0] | 160 | 128.91 | 218.91 |
|  | [0,33,28,30,34,50,0] | 90 | 118.25 | 179.34 |
| RC201.50 | [0,42,39,36,44,41,38,40,35,37,43,0] | 200 | 134.58 | 739.12 |
|  | [0,33,28,27,29,31,30,23,21,18,19,49,22,20,50,34,26,32,24,25,48,0] | 370 | 334.77 | 856.04 |
|  | [0,14,47,16,15,12,11,9,10,13,17,0] | 200 | 127.5 | 753.31 |
|  | [0,5,45,2,6,7,8,46,3,1,4,0] | 190 | 118.12 | 736.13 |
| RC201.100 | [0,92,95,63,33,28,27,29,31,30,62,67,71,61,81,94,96,56,66,0] | 235 | 222.86 | 633.9 |
|  | [0,65,83,64,51,76,85,84,50,34,32,26,89,48,25,77,58,0] | 299 | 253.97 | 833.97 |
|  | [0,5,45,2,6,7,8,46,3,1,4,100,70,0] | 216 | 123.21 | 854.12 |
|  | [0,82,52,12,16,15,11,9,99,57,86,87,97,10,55,68,0] | 313 | 199.32 | 658.09 |
|  | [0,72,36,39,42,44,41,38,40,43,35,37,54,93,91,80,0] | 253 | 164.43 | 866.62 |
|  | [0,14,47,59,75,23,21,18,19,49,22,20,24,74,13,17,60,0] | 280 | 235.62 | 830.21 |
|  | [0,69,98,88,79,73,78,53,90,0] | 128 | 92.21 | 453.08 |
